# Establishment of Recombinant *Eimeria acervulina* Expressing Multi-Copies M2e Derived from Avian Influenza Virus H9N2

**DOI:** 10.3390/vaccines9070791

**Published:** 2021-07-16

**Authors:** Sixin Zhang, Xinming Tang, Si Wang, Fangyun Shi, Chunhui Duan, Feifei Bi, Jingxia Suo, Dandan Hu, Jie Liu, Chaoyue Wang, Xun Suo, Xianyong Liu

**Affiliations:** 1 National Animal Protozoa Laboratory, College of Veterinary Medicine, China Agricultural University, Beijing 100193, China; zhangsixin@cau.edu.cn (S.Z.); wangsi@cau.edu.cn (S.W.); shifangyun@cau.edu.cn (F.S.); chunhuiduan@cau.edu.cn (C.D.); bifeifei@cau.edu.cn (F.B.); suojingxia@cau.edu.cn (J.S.); B20153050195@cau.edu.cn (D.H.); bs20193050493@cau.edu.cn (J.L.); bs20173050413@cau.edu.cn (C.W.); suoxun@cau.edu.cn (X.S.); 2 Institute of Animal Science, Chinese Academy of Agricultural Sciences, Beijing 100193, China; tangxinming@caas.cn

**Keywords:** *Eimeria acervulina*, wing vein, stable transfection, live vaccine vector

## Abstract

The potential of *Eimeria* parasites as live vaccine vectors has been reported with successful genetic manipulation on several species like *E. tenella, E. mitis* and *E. necatrix*. Among seven *Eimeria* species infecting chickens, *E. acervulina* is a highly prevalent, moderately pathogenic species. Thus, it is valuable for the study of transfection and for use as a potential as vaccine vector. In this study, a plasmid containing expression cassette with enhanced yellow fluorescent protein (EYFP), red fluorescent protein (RFP) and 12 copies of extracellular domain of H9N2 avian influenza virus M2 (M2e) protein was used for the transfection. Nucleofected sporozoites were inoculated into birds through wing vein. Recombinant *E. acervulina* oocysts with 0.1% EYFP+ and RFP+ populations were collected from the feces of the inoculated birds. The fluorescent rate of transgenic parasites reached over 95% after nine successive propagations with a pyrimethamine selection in vivo and fluorescent-activated cell sorting (FACS) of progeny oocysts. The expression of M2e in the transgenic parasites (EaM2e) was confirmed by Western blot and its cytoplasm localization in sporozoites was displayed by an indirect immunofluorescent assay (IFA). Meanwhile, we found that the fecundity of EaM2e was equivalent to that of wild type *E. acervulina* (EaWT). Taken together, the stable transfection of *E. acervulina* was successfully established. Future studies will focus on whether transgenic *E. acervulina* can serve as a live vaccine vector.

## 1. Introduction

Coccidiosis is one of the most severe parasitic diseases in chickens, which causes EUR 7.7–13.0 billion losses to the global poultry industry annually [1]. The strategy to control coccidiosis relies on the administration of anticoccidial drugs and live anticoccidial vaccines, the latter of which have been used for 70 years [2,3].

With the development of genetic manipulation technology, genetically modified live vector vaccines are promising vaccines for both humans and animals [4,5]. Since live oocyst-based anticoccidial vaccines are being widely used in the poultry industry, whether *Eimeria* can be genetically manipulated (GM) and used as a vehicle to present protective antigens of other pathogens, such as the avian influenza virus and the infectious bursitis virus. If it can be achieved, GM-anticoccidial vaccines can be used as multivalent vaccines to control coccidiosis as well as infectious diseases caused by other pathogens. Currently, transient and stable transfection of coccidian parasites has been developed in several species of chicken coccidia, including *Eimeria tenella* [6,7,8], *Eimeria mitis* [9,10], and *Eimeria necatrix* [11]. With such success, the potential of *Eimeria* as live vaccine delivery vectors is currently under investigation [12,13]. Transgenic *E. tenella* expressing the immune mapped protein 1 of *Eimeria maxima* or the surface antigen 1 derived from *Toxoplasma gondii* provided immunoprotection against *E. maxima* or *T. gondii* challenge in chickens, respectively [14,15].

As one of the seven chicken *Eimeria* species, *E. acervulina* is characterized by a moderate pathogenesis and a high prevalence in the field. Currently, all available commercial live anticoccidial vaccines contain strains or isolates of *E. acervulina.* Thus, *E. acervulina* could be an ideal vehicle candidate for transgenic *Eimeria-*based vaccine vectors.

Here, we achieved, for the first time, the stable transfection of *E. acervulina* and confirmed the expression of M2e in the whole lifecycle of transgenic *E. acervulina*.

## 2. Materials and Methods

### 2.1. Animals and Parasites

Specific pathogen free (SPF) chickens, 3 weeks old, were purchased from Merial Animal Health Co., Ltd. (Beijing, China). One-day old Arbor Acres (AA) broilers were purchased from Beijing Arbor Acres Poultry Breeding (Beijing, China). All birds were reared in isolators and were fed with coccidia-free diet and water ad libitum.

Oocysts of the *E. acervulina* BJ strain were maintained and propagated in coccidia-free, 2-week-old AA broilers. The oocysts were propagated, collected, sporulated and purified according to previously described methods [16]. Briefly, 1000 sporulated oocysts per bird were orally inoculated. Fresh oocysts in the feces were collected by flotation in saturated salt solution 5–7 days post inoculation, and incubated in 2.5% (*w*/*v*) potassium dichromate at 27 °C for 24–48 h. After sporulation, the oocysts were sterilized and purified with sodium hypochlorite followed by quantification using a McMaster counting chamber (CAAS Shanghai Vet Institute, Shanghai, China) and maintained in 2.5% (*w*/*v*) potassium dichromate at 4 °C.

### 2.2. Plasmid Construction and In Vivo Transfection of E. Acervulina

**Figure 1:**
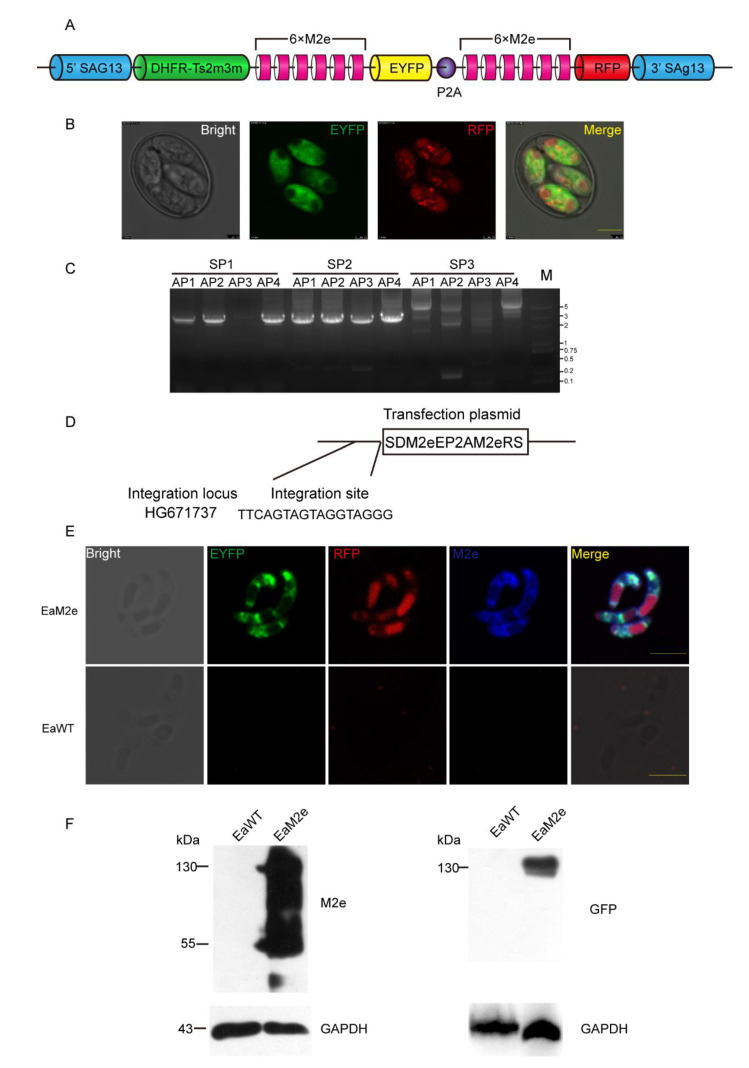
Construction and identification of transgenic *E. acervulina* expressing multiple copies of M2e. (**A**) Schematic diagram of the transfection plasmid containing 12 copies of M2e. The expression cassette co-expressing 12 copies of M2e was constructed by fusing each fluorescent gene with six copies of M2e and linked by P2A sequence. (**B**) The expression pattern of EYFP and RFP in the sporulated oocysts of EaM2e. (**C**) Genomic DNA from EaM2e was amplified with arbitrary degenerate primers (AP1, AP2, AP3, and AP4) from the genome walking kit and specific primers (SP1, SP2 and SP3) from *sag 13* 5′ UTR by three rounds of thermal asymmetric interlaced PCR, and gel electrophoresis of amplified products. M: DL plus 2000 marker. (**D**) Integration site of the exogenous plasmid into *E. acervulina* genome was analyzed by BLAST in the *E. acervulina* DB database according to sequence results. (**E**) The distribution of EYFP, RFP, and M2e in the EaM2e sporozoites. M2e distribution was detected by IFA, which used the mouse anti-M2e polyclonal antibodies and the AMCA-conjugated goat anti-mouse IgG as primary and secondary antibodies, respectively. Bar = 5 μm. (**F**) Western blot analysis of the expression of DHFR-M2e-EYFP and M2e-RFP fused protein in the transgenic *E. acervulina* lines. Polyclonal antibodies against GFP (1:2000), polyclonal antibodies to M2e (1:5000,) and GAPDH of *E. tenella* from mouse (1:200) were used as the primary antibodies. Antigens from EaWT served as the controls.

The construction of the plasmid, pSDM2eP2AM2eRS, used for the transfection of *E. acervulina* was based on the pSDEP2ARS plasmid [17]. M2e is the extracellular domain of the M2 protein of the H9N2 subtype avian influenza virus (HK strain). The tandem-repeated 6 copies of M2e fragments were synthesized by Beijing Ruibiotech Co., Ltd (Beijing, China). Two tandem M2e fragments, ligated with in-frame EYFP and RFP genes, and DHFR-Ts2m3m (a gene resistant to pyrimethamine) derived from *T. gondii* [8] were under the regulation of a surface antigen 13 (*sag13*) promoter and a 3′ regulatory sequence from *E. tenella* in the expression cassette. A porcine teschiovirus-1 2A peptide (P2A, 66 bp), which was demonstrated to be able to cleave two contiguous proteins [17,18], was inserted between EYFP and M2e (Figure 1A). The maxi-prepared plasmid was linearized with a S*naB* I restriction enzyme before the transfection.

For the stable transfection of *E. acervulina*, a restriction enzyme-mediated integration method was used [19]. The sporozoites were purified with DE-52 cellulose column [20,21], then transfected with linearized plasmid DNA and then inoculated into four 3-day-old chickens via the wing vein (1.0 × 10^7^ sporozoites/bird). The oocysts were collected from the feces between days 5 and 7 post-inoculation. The oocysts expressing EYFP and RFP were FACS sorted (MoFlo, Dako Cytomation, Fort Collins, CO), and each bird was inoculated with 1000 sorted oocysts. To further screen the transgenic *E. acervulina*, pyrimethamine (150 mg/kg feed) was added to the feed until the fluorescent rate of oocysts was more than 95% in the population.

### 2.3. Genome Walking

To confirm the integration of the exogenic DNA fragment into the genome of transgenic *E. acervulina*, the flanking sequences of the 5′ integration site were identified using a genome walking kit (Takara, Dalian, China). The extraction and validation of genomic DNA from transgenic *E. acervulina* was performed according to the previously described methods [9]. Specific primers (SP) were designed according to *E. tenella Sag13* promoter sequence as previously described [17]. PCR products of the third round were recovered and cloned into the pEASY-T1-simple vector (TransGen Biotech, Beijing, China). The sequencing results were analyzed by DNAStar7.0 software, and the integration sites in the genome were identified by performing a BLAST search in the *E. acervulina* DB database [22].

### 2.4. IFA

To validate the location of M2e in the transgenic *E. acervulina* sporozoites, IFA was conducted as previously described [10]. Briefly, the sporozoites of EaM2e or EaWT were air-dried on slides with poly-L-lysine, fixed with 100% methanol and permeabilized with 0.2% Triton X-100. Then, the slides were blocked with 1% BSA, and probed with mouse anti-M2e polyclonal antibodies (1:1000 dilution) at 37 °C for 1 h. After incubation with secondary antibodies, aminomethylcoumarin acetate (AMCA)-conjugated goat anti-mouse IgG (Proteintech, 1:200 dilution) at 37 °C for 1 h, the slides were sealed with nail polish and observed using a confocal microscope (SP5, Leica, Germany).

### 2.5. Western Blot

The collection of the second generation of schizoites was carried out as previously described [11,23]. Briefly, the 2-week-old broilers were sacrificed 72 h after EaM2e or EaWT infection. The duodenum was cut longitudinally, rinsed three times with PBS, and dissected into sections (5 mm). The samples were incubated with a digestion buffer (PBS containing 0.25% trypsin and 0.5% sodium taurodeoxycholate hydrate) at 37 °C for 30 min. The suspension was filtered with gauze and centrifuged at 1500 rpm for 10 min to collect the schizoite pellet. For the Western Blot assay, soluble proteins from the purified EaM2e or EaWT sporozoites or schizonts were prepared as previously described [24]. For the detection of M2e and EYFP, the PVDF membranes were probed with mouse anti-M2e polyclonal antibodies (1:5000) and rabbit anti-GFP polyclonal antibodies, which were also probed by the YFP (Proteintech, 1:3000) at 37 °C for 1 h. The HRP-conjugated goat anti-rabbit and goat anti-mouse secondary antibodies were used for detection. The Glyceraldehyde-3-Phosphate Dehydrogenase (GAPDH) or Actin of *E. acervulina* was used as an internal reference.

### 2.6. Immunohistochemistry Assay

The duodenum of the 2-week-old AA broilers was sampled every 12 h, from 48 h to 120 h after infection with transgenic parasites. The tissue section preparation and H&E staining were carried out as previously described [25]. The sections were deparaffinized with xylene and treated with serial dilutions of ethanol, and the antigen retrieval was performed by pressure cooking method [26]. Then, the slides were sequentially permeabilized with 0.5% Triton X-100, blocked with 10% goat serum, and incubated with anti-M2e polyclonal antibodies (1:500) at 4 °C overnight. After incubation with the second antibodies, Cy3-conjugated goat anti-mouse IgG at 37 °C for 1 h, and stained with DAPI for 7 min, the slides were observed using a confocal microscope.

### 2.7. Measurement of the Fecundity of Transgenic Parasite EaM2e

Four groups of 3 SPF chickens were each orally inoculated with 500 or 1000 sporulated oocysts of EaWT or EaM2e, respectively. Between days 3 and 14 post-inoculation, the oocysts in the feces were collected and counted every 24 h using a McMaster chamber.

### 2.8. Test of Antibody Titer Elicited in Chickens Immunized by Transgenic EaM2e-Expressed M2e Peptides

Three groups of 6 SPF chickens were set as the control (Ctrl) EaWT and EaM2e groups. The immunization of the chickens was performed three times with an interval of two weeks. Each chicken was vaccinated with 5 × 10^2^, 5 × 10^3^, and 5 × 10^4^ oocysts of EaWT or EaM2e, respectively. The control group was inoculated with 200 μL PBS each time. The chicken serum samples were collected 1 day before the primary immunization and 2 weeks after each immunization. Enzyme-linked immunosorbent assay (ELISA) was performed as previously described [10]. The plates were coated with *E. acervulina* soluble antigens (200 ng/well). The chicken sera (1:100 dilution) and HRP-conjugated goat anti-chicken IgY Fc fragment (1:5000 dilution, Bethyl Laboratories, Inc., Montgomery, AL, USA) were used as the primary and secondary antibodies, respectively.

### 2.9. Statistical Analysis

Data were statistically analyzed by the Mann–Whitney U test and Dunnett’s multiple comparisons test using GraphPad Prism 8.0 (GraphPad Software, San Diego, CA, USA). The differences between groups were considered significant at *p* < 0.05.

## 3. Results

### 3.1. Establishment and Validation of a Stably Transfected Line of E. acervulina

Through the inoculation of transfected sporozoites of *E. acervulina* into the wing vein, we obtained the progeny oocysts expressing dual fluorescent reporter genes EYFP and RFP (Figure 1B). Combined with FACS sorting and pyrimethamine selection, a stable transfection line of *E. acervulina*, EaM2e, was established after nine successive propagations. The EaM2e reached over 95% fluorescent rate without drug selection and FACS after the 10th propagation (Table 1).

To validate the insertion of exogenous genes into the genome of *E. acervulina*, the analysis of genomic DNA of transgenic *E. acervulina* was performed through genome walking (Figure 1C). Sequencing results showed that 5′ ends of the exogenous plasmid were integrated into the HG671737 locus (Figure 1D). To evaluate the location and expression pattern of the exogenous genes in the transgenic parasites, we performed an indirect immunofluorescence assay (IFA). In the EaM2e sporozoites, EYFP is expressed in the cytoplasm while RFP is expressed in the retractable body (Figure 1E). The location of M2e in the EaM2e is in the cytoplasm of sporozoites when being detected with IFA (Figure 1E). The expression of M2e was also confirmed by Western blot, with results showing two specific bands of approximately 130 kDa and 55 kDa, which are consistent with the sizes of DHFR-Ts-6 × M2e-EYFP and 6 × M2e-RFP proteins, respectively (Figure 1E). Collectively, we obtained a stable transgenic *E. acervulina* expressing multi-copies of M2e through the inoculation route of wing vein.

### 3.2. Detection of M2e Expression during the Endogenous Development Stage of EaM2e

**Figure 2:**
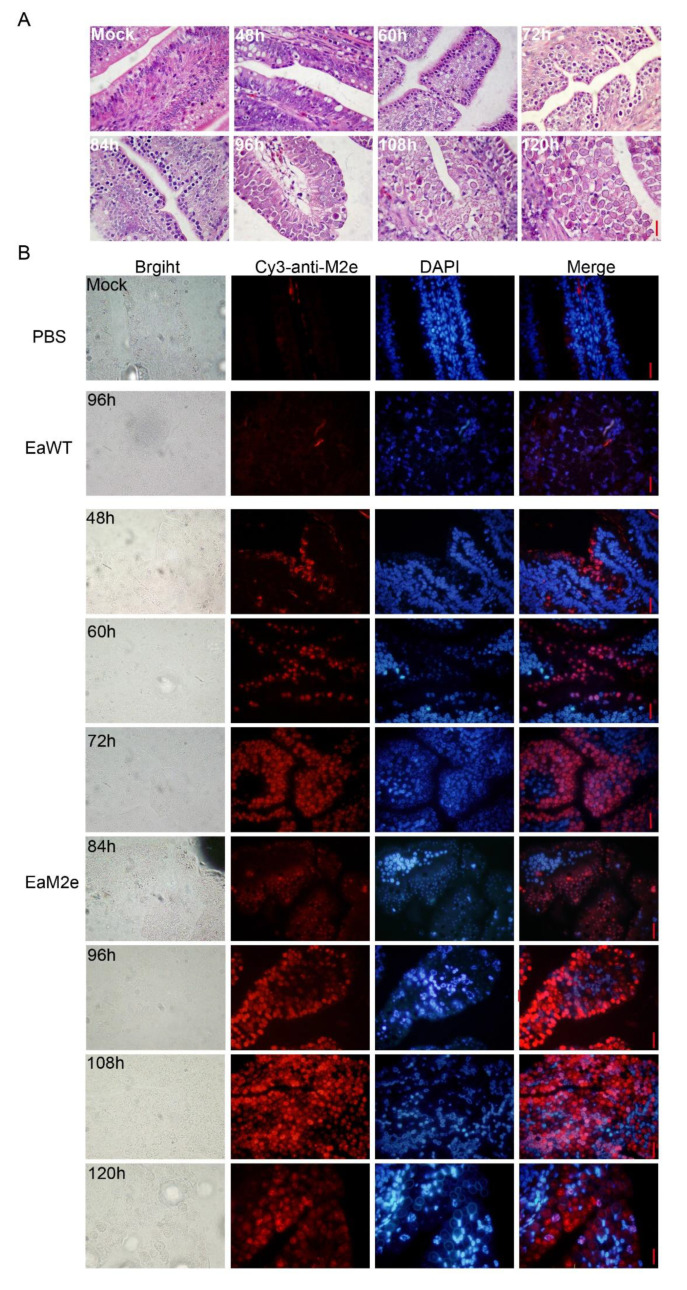
The expression of M2e in the endogenous stage. (**A**) H&E staining of the duodenum of chickens 48 h to 120 h after the EaM2e infection bar = 20 μm. (**B**) Immunofluorescence for M2e of the duodenum of chickens 48 h to 120 h after the EaM2e infection, mouse anti-M2e polyclonal antibodies (1:500) were used as the primary antibody, Cy3-goat anti-mouse IgG (1:200) as the second antibody. The nucleus was stained with DAPI bar = 20 μm.

**Figure 3:**
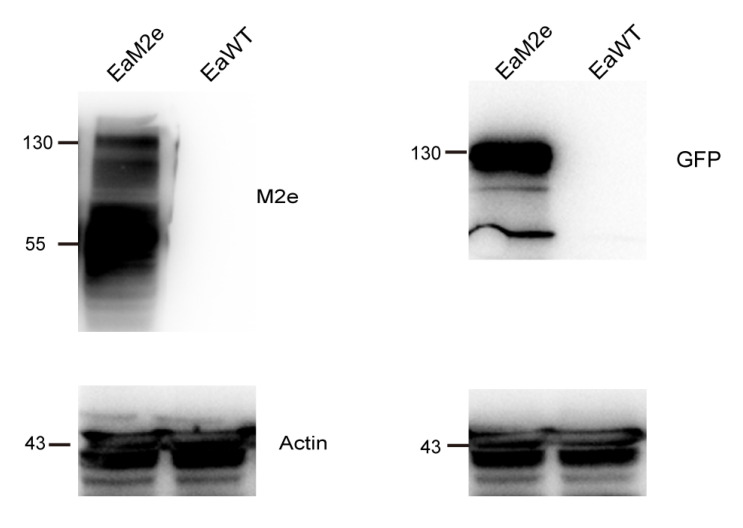
Western blot analysis of M2e protein expression in second-generation schizoites. Polyclonal antibodies against GFP (1:2000), mouse anti-M2e polyclonal antibodies (1:5000), and anti-*T. gondii* actin polyclonal antibodies (1:5000) were used as the primary antibody. Antigens from EaWT served as the controls.

To detect the expression of M2e in the endogenous development, tissue sections of the duodenum of the EaM2e infected birds were subjected to H&E and immunohistochemistry. Numerous second-generation merozoites appeared at 72 h post inoculation, while gametogony-stage parasites and immature oocysts were observed from 96–120 h (Figure 2A). Similarly, the expression of M2e was detected in the merozoites and gametocytes of EaM2e, while there was no response to the M2e antibodies in the tissue section of the PBS and EaWT infected birds (Figure 2B). The Western blot test also showed that M2e is expressed in the second generation merozoites (Figure 3). The above results showed that the M2e was constitutively expressed in the endogenous development stage.

### 3.3. Reproduction of EaM2e

**Figure 4:**
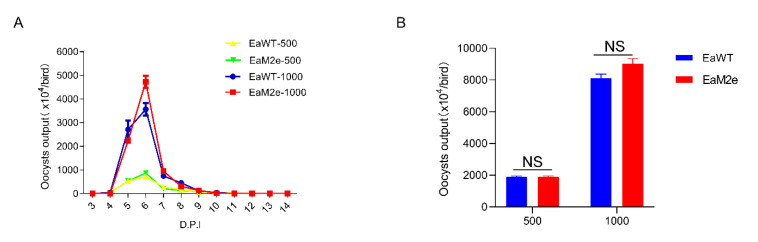
Comparison of the oocyst shedding pattern and fecundity of EaM2e with that of EaWT. (**A**) The oocyst shedding patterns of transgenic *E. acervulina* and the wild type. (**B**) The fecundity of EaM2e was measured after inoculation with 500 or 1000 sporulated compared with those of EaWT. Each value represents the mean ± SD of three birds. Groups with different treatments were analyzed by the two-tailed non-parametric Mann–Whitney U test; NS: not significant.

Next, we explored the biological characteristics of the transgenic *E. acervulina* parasites including the oocyst output curve and the total oocyst output. The transgenic EaM2e showed a similar pattern of oocyst shedding to that of wild type *E. acervulina* (EaWT), with a peak of oocyst output at 6 d after inoculation (Figure 4A). The total yield of EaM2e was similar to that of EaWT when birds were inoculated with an equivalent dose of sporulated oocysts, and no significant difference was found (Figure 4B). These results showed that the insertion and expression of M2e had no obvious effect on the reproduction of *E. acervulina*.

### 3.4. EaM2e Induced Equivalent IgY Antibody Compared with EaWT

**Figure 5:**
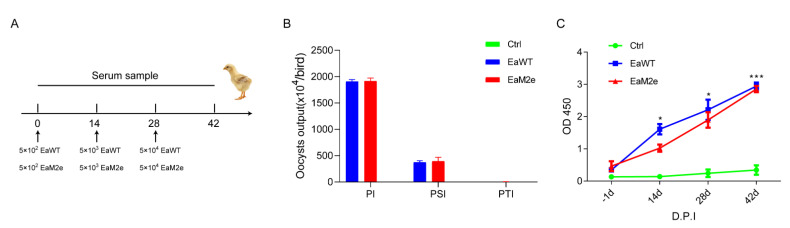
The humoral immune response of chickens to *Eimeria*-soluble antigens after oral vaccination. (**A**) Schematic illustration of the vaccination. (**B**) Oocyst outputs after each immunization. PI: primary immunization; SI: second immunization; TI: third immunization. (**C**) *Eimeria*-specific IgY antibodies were detected by ELISA after −1 d, 14 d, 28 d and 42 d. The data with immunization treatment were compared with that of the Ctrl group and analyzed using Dunnett’s multiple comparisons test (*: *p* < 0.05; ***: *p* < 0.001).

To explore the humoral response elicited by transgenic *Eimeria*, chickens were immunized three times with parasites and chicken serum samples were collected (Figure 5A). Oocyst outputs of EaM2e were similar to that of EaWT after each immunization (Figure 5B). The IgY antibody specific to the soluble antigens of *E. acervulina* could be elicited by vaccination with either EaM2e or EaWT (Figure 5C).

## 4. Discussion

Vaccines play a major role in controlling infectious diseases in animals and humans. With the development of genetic manipulation, the feasibility of *Eimeria* as a carrier for live vaccines has been thoroughly studied, especially in *Eimeria tenella.* Here, we successfully established, for the first time, a stable transgenic *E. acervulina* population by the wing vein inoculation route.

Significant progress has been made in the biology and immunology of Apicomplexan parasites with the development of genetic manipulation. Because *Eimeria* spp. cannot complete their lifecycle or continuously culture in vitro [27], the genetic manipulation must be performed in vivo and the inoculation route is an essential step to obtain transgenic parasites. Depending on the parasitic site, different inoculation strategies are adopted. For the parasitism in the lower part of the intestine such as cecum, it is convenient to operate the transfection via the rectum inoculation [8,9]. For the upper intestine, the most effective method is to inject to the parasitic site by surgery, which is also feasible when stomach acid is neutralized with sodium bicarbonate [8,28,29,30]. *E. acervulina* parasitizes in the duodenum of the chicken, so the inoculation route via the cloaca applied to *E. tenella and E. mitis* was not feasible in *E. acervulina*. Meanwhile, previous studies have found that oocysts can be collected from feces by intravenous inoculation of sporozoites [31] and this is a very important clue to the transfection of *E. acervuline* that allowed us to successfully obtain the progeny oocysts via the inoculation the wing vein route. It also provides an alternative way to obtain transgenic parasites with predilection sites close to the upper intestine.

The IFA results showed that M2e is not co-located in the refractile body with RFP, while it is co-located in the cytoplasm with EYFP. This may be related to the particularity of the refractive body. Meanwhile, the function of the “self-cleaving” 2A peptide was also efficient in the transgenic *E. acervulina*, according to the results from Figure 1E,F. The oocyst production of this transgenic *E. acervulina* is an important biological feature of *Eimeria* spp., compared with the wild type. We found that the transgenic parasites produced more oocysts than the wild type, which was consistent with previous study on transgenic *Eimeria tenella* expressing M2e [32], whereas many studies have found that the fecundity of transgenic *Eimeria* was weaker than wild type *Eimeria* [7,9,14,29]. We speculated that this may be related to the insertion site of M2e in the genome or that the expression of M2e affected the reproduction of *E. acervulina.* Due to the failure to obtain a monoclonal population of transgenic *E. acervulina*, the underlying mechanism needs further study.

The M2e is highly conserved across all influenza A isolates and has become an ideal candidate for the development of an effective cross-protective vaccine against influenza. However, Liu et al. found that the immune responses elicited by the transgenic *E. tenella* expressing monomer M2e were not good [32]. Ma et al. showed that four copies of M2e induced strong M2e-specific IgG antibodies and limited viral replication [33]. Therefore, we constructed a transgenic *E. acervulina* line that expresses twelve copies of M2e. Although the M2e was expressed the whole life cycle, the immune response, including the antibodies titer against to M2e and IFN-γ secretion lymphocytes (data not showed) induced by transgenic *E. acervulina*, was not significantly different from the control group. We reasoned that the location of the M2e expression may be responsible. Some researchers have found that secreted antigen or surface antigen induced stronger immune responses than cytoplasmic proteins [34,35]. If M2e is displayed on the surface or secreted, a stronger immune response may be induced.

At present, although we have achieved stable transfection of *E. acervulina*, how to improve the immune response of heterologous pathogen antigens is still a problem. Due to the low transfection efficiency, we mainly combined FACS sorting and drug screening to improve the fluorescent rate of transgenic *E. acervulina*. Now we have achieved transgenic *E. acervulina* with a fluorescent rate of more than 95% by using only fluorescent protein (EYFP) as a screening marker (Yu et al., unpublished data). At the same time, our laboratory has also achieved CRISPR/Cas9 gene knockout on *Eimeria* [36,37]. Therefore, after obtaining a stable transgenic *E. acervulina* line, we can use a gene-editing system to knock out the other selection markers like YFP, RFP, and DHFR-TS.

In the future, once it is realized that *Eimeria* can be used as a vaccine carrier to present protective antigens from heterologous pathogen, if the immune protection effect is enough to resist heterologous pathogens, the immunity of chickens can be built only via vaccination with live vector vaccine through drinking water, which is convenient and has less stress response than intramuscular inoculation. If there is only a certain degree of immune protection, we can combine live vector vaccine and pathogen-specific vaccine as a prime-boost vaccine strategy to build immunity.

## 5. Conclusions

Collectively, we successfully established the stable transfection of *E. acervulina* via the inoculation route of the wing vein. Our findings will encourage the development of transgenic *E. acervulina* as a live vaccine vector for use in birds.

## Figures and Tables

**Table 1:** Propagation of transgenic *E. acervulina*.

Generation	Percentage of Fluorescent Oocysts (%)	Selection Strategy
1st *	~0.1	--
2nd	2.63	Drug + FACS
3rd	15.28	Drug + FACS
4th	31.2	Drug + FACS
5th	32.3	Drug + FACS
6th	35.2	Drug + FACS
7th	65.4	Drug + FACS
8th	92.4	Drug + FACS
9th	>95	Drug + FACS
10–12th	>95	--

*: Inoculation with nucleofected sporozoites, while inoculation with sorted oocysts was performed for other generations.

## Data Availability

All the raw data is available and provided upon request.
